# Genome-Wide Characterization of RNA Editing Sites in Primary Gastric Adenocarcinoma through RNA-seq Data Analysis

**DOI:** 10.1155/2020/6493963

**Published:** 2020-12-18

**Authors:** Javad Behroozi, Shirin Shahbazi, Mohammad Reza Bakhtiarizadeh, Habibollah Mahmoodzadeh

**Affiliations:** ^1^Department of Medical Genetics, Faculty of Medical Sciences, Tarbiat Modares University, Tehran, Iran; ^2^Department of Animal and Poultry Science, College of Aburaihan, University of Tehran, Tehran, Iran; ^3^Department of Surgical Oncology, Cancer Institute, Imam Khomeini Hospital Complex, Tehran University of Medical Sciences, Tehran, Iran

## Abstract

RNA editing is a posttranscriptional nucleotide modification in humans. Of the various types of RNA editing, the adenosine to inosine substitution is the most widespread in higher eukaryotes, which is mediated by the ADAR family enzymes. Inosine is recognized by the biological machinery as guanosine; therefore, editing could have substantial functional effects throughout the genome. RNA editing could contribute to cancer either by exclusive editing of tumor suppressor/promoting genes or by introducing transcriptomic diversity to promote cancer progression. Here, we provided a comprehensive overview of the RNA editing sites in gastric adenocarcinoma and highlighted some of their possible contributions to gastric cancer. RNA-seq data corresponding to 8 gastric adenocarcinoma and their paired nontumor counterparts were retrieved from the GEO database. After preprocessing and variant calling steps, a stringent filtering pipeline was employed to distinguish potential RNA editing sites from SNPs. The identified potential editing sites were annotated and compared with those in the DARNED database. Totally, 12362 high-confidence adenosine to inosine RNA editing sites were detected across all samples. Of these, 12105 and 257 were known and novel editing events, respectively. These editing sites were unevenly distributed across genomic regions, and nearly half of them were located in 3′UTR. Our results revealed that 4868 editing sites were common in both normal and cancer tissues. From the remaining sites, 3985 and 3509 were exclusive to normal and cancer tissues, respectively. Further analysis revealed a significant number of differentially edited events among these sites, which were located in protein coding genes and microRNAs. Given the distinct pattern of RNA editing in gastric adenocarcinoma and adjacent normal tissue, edited sites have the potential to serve as the diagnostic biomarkers and therapeutic targets in gastric cancer.

## 1. Introduction

RNA editing is a common and essential posttranscriptional alteration of RNA sequences, affecting millions of bases, expanding the transcriptome diversity and the functions of RNA transcripts [1]. Although several types of RNA editing have been characterized, conversion of adenosine residues to inosine (A to I) is the most frequent type of editing in humans. This reaction is catalyzed by the double-stranded RNA- (dsRNA-) specific adenosine deaminase that acts on the RNA (ADAR) family [2, 3]. Resulting inosine is recognized by most of the biological machinery as guanosine (G); consequently, editing could have a protein recoding outcome, generating proteomic and phenotypic diversity [4, 5].

RNA editing diversifies the transcriptome when editing located in coding mRNA sequences; at the same time, editing in the noncoding sequence could have a fundamental consequence. 3′UTRs usually comprise key elements and are involved in numerous regulatory processes. Editing in these elements can modulate the regulation of mRNA expression [3]. MicroRNAs identify their target genes primarily by sequence complementarity between the microRNA seed region and a target site; hence, editing in the seed sequence could affect target recognition [6]. Indeed, editing in the nonseed sequence may alter microRNA structure or stability, leading to biological consequences. It is also believed that editing of precursor microRNA may inhibit its processing to mature microRNA [7].

RNA editing is critical for growth and development in mice and humans. Hence, knockout mice for either ADAR or ADARB1 genes die early in development. Interestingly, the exonic substitution of an underedited transcript of the AMPA receptor gene with the edited version could rescue ADARB1-knockout mice [8, 9].

ADAR gene mutations are associated with several human diseases. Aicardi-Goutieres syndrome (AGS; OMIM #225750) [10] and dyschromatosis symmetrica hereditaria (DSH; OMIM #127400) are correlated with the mutations in the ADAR deaminase domain [10]. The transcriptome of nearly all normal cell types is actively edited, particularly in the immune system and the central nervous system, which exhibit fundamental flexibility of function. On the other hand, editing seems to be decreased in static cells, such as muscle cells, where there is no need for novel adaptations [11]. It has been reported that RNA editing events are a positive contribution to cancer development and progression [3]. RNA editing dysregulation has been linked to cancer by either editing in coding [12, 13] or noncoding [14, 15] sequences. There is a strong consensus on the effect of global editing levels in cancer, and increased genome-wide editing rates have been reported in some cancers including breast tumors, head/neck squamous cell, thyroid and lung adenocarcinoma, and kidney renal cell carcinomas. Conversely, decreased whole editing rates were seen in kidney chromophobe and renal papillary carcinoma [16]. RNA editing can exert anti- and protumorigenic activities through the regulation of immune responses. In some tumors, increased levels of edited peptides, such as cyclin I, lead to the antigen-specific killing of tumor cells through cytotoxic T cells [17]. On the contrary, hyperediting of inverted repeat elements promotes immune silencing and tumor viability [18].

ADAR proteins bind to a specific dsRNA structure formed either intramolecularly or intermolecularly; thus, ADAR particularly edits A to I on RNAs that adopt this double-strand structure [19]. There are also some modifying features including RNA sequence preference associated with neighbor editing sites [20], editing inducer elements distant from editing position [21], and base opposing the edited adenosine [22]. Despite the identification of these regulatory elements, the main controlling feature of ADAR target recognition and how the ADAR nominates an adenosine for edition remain to be further studied. Since these elements do not allow the prediction of editing sites, identification of editing events is therefore dependent on sequencing data [23].

The advent of next-generation sequencing (NGS) has greatly improved the genome-wide identification of RNA editing sites through RNA sequencing (RNA-seq) technologies, and so far several million high-confidence editing sites have been recognized in the human genome [24]. Identification of editing sites from RNA-seq data seems to be straightforward. Simply, aligning RNA-seq reads to the reference genome and searching for A to G mismatches lead to detection of editing sites [25]. However, there are several sources of disagreement between the RNA sequence and the reference genome, making the identification of actual editing sites challenging. Using RNA-seq data to identify RNA editing events comprises a major challenge which is the discrimination of genuine editing sites from somatic mutations, SNPs, and sequencing errors. Robust bioinformatical approaches need to overcome such a drawback [26]. However, dozens of outstanding studies have successfully employed RNA-seq data alone to identify editing events [26–35].

RNA editing has been studied in gastric cancers [36]; nevertheless, many questions on the extent and consequences of RNA editing in gastric cancer remain concealed. In this study, we leveraged publicly available sequencing datasets to characterize RNA editing in gastric cancer.

## 2. Materials and Methods

### 2.1. RNA-seq Datasets

Raw paired-end RNA-seq samples related to eight primary gastric adenocarcinoma and their paired nontumor counterparts were retrieved from the publicly available Gene Expression Omnibus (GEO) database (accession number GSE85465). Nontumor counterparts refer to samples harvested from the stomach, from sites distant from the tumor and exhibiting no visible evidence of tumor or intestinal metaplasia/dysplasia upon surgical assessment. The original data and sample details are described by Ooi et al. [37]. RNA-seq libraries of these samples were constructed using the Illumina Stranded Total RNA Sample Prep Kit v2, and the dataset was generated using the Illumina HiSeq 2000 platform and the paired-end 101 bp read option.

### 2.2. Quality Control and Read Mapping

First, FastQC v0.11.5 (http://www.bioinformatics.babraham.ac.uk/projects/fastqc/) was employed to control the raw read quality [28]. Sequencer adapter removal and quality trimming were performed by using Trimmomatic v0.32 (parameters: trailing 20 Maxinfo 60:0.95 and minimum length 60) [38]. Then, clean reads were aligned to the human reference genome (GRCh38) using Hisat2 v2.0.5 [39]. To reduce the potential bias caused by short read alignment, only uniquely and concordantly aligned reads were kept.

Using the MarkDuplicates tool from the Picard package (http://picard.sourceforge.net/), PCR-induced duplicate reads mapped to the same location were excluded, except those with the highest mapping quality score. To improve the quality of reads and the alignment of the indel flanking regions, the remaining reads were locally realigned around putative indels and the base quality values were recalibrated by using the GATK tool v3.5 (https://www.broadinstitute.org/gatk).

### 2.3. Variant Calling and Identification of RNA Editing Sites

Variant calling was performed using the HaplotypeCaller from the GATK tool. RNA-DNA differences (RDDs) were called with stand call_onf and stand_emit_conf values of 30 and mbq of 25 [40]. Next, the RDDs were removed from further analysis if they corresponded to known SNPs found in the Ensembl human SNP database version 151. Then, the remaining variants were filtered using the GATK standard filters including (1) total depth of coverage < 10, to remove variants with less than 10 reads that passed the caller's internal quality control metrics; (2) HomopolymerRun > 5, to eliminate the variants with a homopolymer run larger than 5 bp on either side; (3) RMSMappingQuality < 40, to exclude variants with root mean square mapping quality less than 40 over all the reads at the site; (4) MappingQualityRankSum < −12.5, which compares the mapping qualities of the reads supporting the reference allele and the alternate allele and was employed to avoid mapping quality bias—a negative value indicates that the mapping qualities of the reference allele are higher than those supporting the alternate allele; (5) QualitybyDepth < 2, which is intended to normalize the variant confidence in order to avoid inflation caused when there is deep coverage; and (6) ReadPosRankSum < −8, which compares whether the positions of the reference and alternate alleles are different within the reads and eliminate variant distance bias [41]. Employment of GATK tools has been validated in identifying RNA editing sites from RNA sequencing data, without the need for a matched genomic DNA sequence [26].

**Figure 1:**
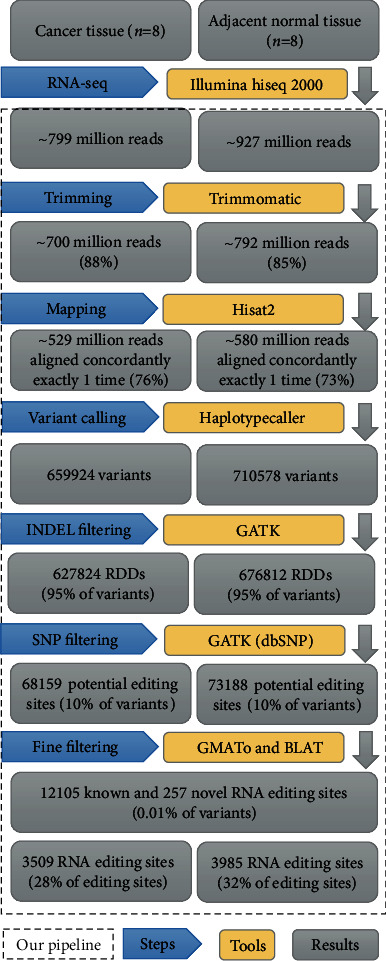
Bioinformatical approach used for the identification of RNA editing sites in normal and cancerous gastric tissue from RNA-seq datasets.

Additionally, several quality-aware filtering steps were employed to increase the accuracy of identifying true RNA editing sites. First, the sites with more than one nonreference type and homozygous sites for the alternative allele were filtered. Second, the sites with fewer than three RDD supporting reads were discarded, and the sites with at least 10-read coverage were kept for further analysis. The RDD sites with an extreme or a rare degree of variation (the threshold for the editing ratio was between 10% and 90%) were removed under the assumption that 100% editing efficiency is unrealistic. Third, RDDs located in regions with bidirectional transcription (transcription that occurs on both the positive and negative strands) were also filtered. Fourth, the GMATo software was used for the detection of simple sequence repeat (SSR) patterns. RDDs located in SSR regions were considered biased with an offset of ±3 bases [42], and those within the 5 bp intronic flanking region were removed. Finally, to reduce false-positive RDDs because of misalignment of sequencing reads to other parts of the genome, we filtered out RDDs in paralogs or repetitive regions by retrieving and aligning 100 bp of the flanking sequence (50 upstream and 50 downstream of the RDD) using BLAT [43]. Only the RDDs that were located in uniquely mapped sequences were considered RNA editing sites. A to G and editing sites were kept for further analysis, and other noncanonical editing sites were excluded. The functional annotation and genomic location of the RNA editing sites were performed using SnpEff v4.3 [44]. The gene set used for annotation was Ensembl version GRCh38.92. Ultimately, we compare identified RNA editing sites with those in DARNED, RADAR, and REDIportal [45–47] databases and categorized them as “known RNA editing sites,” if they were in these databases, and as “novel editing sites” if they were not. An overview of our computational analysis pipeline for identifying the RNA editing sites is shown in Figure 1.

### 2.4. Neighborhood Profile of Editing

In order to predict the conservation of the editing sites, neighborhood nucleotides, 10 bp upstream and 10 bp downstream of the edited sites, were extracted. Then, the WebLogo software was employed to generate a consensus sequence logo and investigate the sequence context flanking the identified potential editing sites [48].

### 2.5. In Silico Validation of Detected Editing Sites

To validate detected RNA editing sites, we applied the publicly available human expressed sequence tags (ESTs) (ftp://ftp.ncbi.nih.gov/repository/UniGene/) to investigate the presence of the editing events identified by our pipeline in these sequences. Using BLAST, 50 bp upstream and downstream flanking regions of editing sites were extracted and queried against the human EST sequences. Alignments with *e*-values < 10^−5^ were considered significant and counted. Since most of A to I RNA editing occurs in Alu repeats [49], we evaluate the intersection of Alu repeats with identified editing sites. To do this, genomic positions of Alu repeats were downloaded from the UCSC database (http://genome.ucsc.edu/) and their distribution patterns across the genome were compared with the patterns of identified A to I editing sites.

### 2.6. Confirmation of Editing Sites by Sanger Sequencing

Validating a small number of edits across few samples is a limitation of the study which warrants further investigation. RT-PCR and Sanger sequencing were performed to validate 11 editing sites on the last exon of the NOP14 gene. RNA and DNA were isolated from normal and cancer tissues of 42 gastric cancer patients, using the All-In-One DNA/RNA Miniprep Kit (Biobasic, Canada). Nucleic acid quality and quantity were verified by using 1% agarose gels and using the NanoDrop spectrophotometer (NP80, Germany). RNA samples were reverse transcribed into cDNA using the Easy™ cDNA Synthesis Kit (Parstous Biotechnology, Iran). Amplification was performed by using an XP thermal cycler (Bioer, China). Cycling conditions were as follows: 94°C for 5 min, 10 cycles of 98°C for 10 s, 67°C for 10 s with a decrement of 1°C every cycle, and 72°C for 35 s, and then 23 cycles of 98°C for 10 s, 57°C for 10 s, and 72°C for 35 s. The primers were designed to amplify products of an appropriate size for gDNA and cDNA sequencing. The identified editing sites were considered confirmed if the cDNA sequence was heterozygous while the corresponding DNA sequencing was homozygous. The ImageJ software was used for quantification of sequencing data [50].

### 2.7. Editing of MicroRNA Target Sites

In order to predict microRNAs whose binding is affected by RNA editing, we downloaded the predicted microRNA binding data of highly conserved miRNA families from the miRcode database [51]. The intersect feature of BEDTools was then applied to find RNA editing sites that overlap with the target site of microRNAs [52].

### 2.8. Statistical Analyses

Statistical significance for differences between cancer and normal tissue editing ratios was assessed by the Wilcoxon rank test. Spearman's correlation coefficient was used to determine the relationship between chromosome length, number of Alu elements, number of protein coding genes, and number of editing sites. Differences were considered significant when the adjusted *P* value was <0.05.

## 3. Results

### 3.1. Identification of RNA Editing Sites

**Figure 2:**
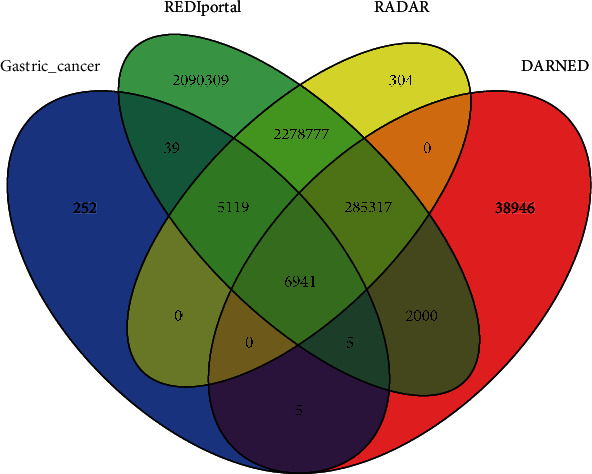
Venn diagram of identified editing sites in our study and three other databases.

**Figure 3:**
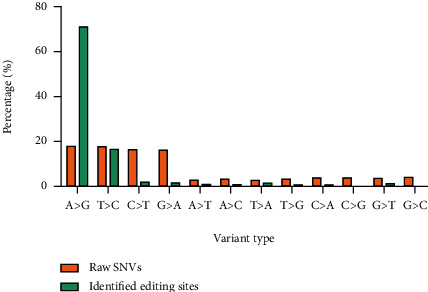
Percentage of all twelve mismatch types.

High-throughput RNA-seq technology has facilitated the discovery of transcriptome-wide RNA editing events across individuals and tissues at unprecedented throughput and resolution. However, the main obstacle in identifying bona fide RNA editing sites using RNA-seq data is the distinction of RNA editing sites from rare SNPs and technical artifacts caused by sequencing or read mapping error. To accurately detect the RNA editing sites at the transcriptome-wide level in gastric cancer, we developed a computational approach by using a precise strategy (see Figure 1). This strategy enabled us to identify the potential RNA editing sites using RNA sequencing data alone, without the need for the available matched DNA sequence from the same sample. We obtained 1725 million reads from RNA-seq data of eight gastric adenocarcinoma and their paired normal tissues. After quality trimming, a total of 1492.1 million reads were generated from all samples (on average, 93.3 million reads per sample). The clean reads were aligned to the reference genome with an average mapping rate of 91.67%. The average rate of uniquely and concordantly mapped reads was 74.34% (range 59-84%). An initial analysis led to the identification of 1370502 variants, and after excluding SNPs and indels, 141347 RDDs remained. By applying multiple stringent filters to exclude false positives, a total of 12362 unique A to G RNA editing sites were identified across all samples. Of which, 12105 sites were previously reported in the RNA editing database and 257 variants were novel editing sites (Figure 2). More than 70% of identified final editing sites were A to G events, while 18% of raw RDDs were related to A to G variants (Figure 3). These editing sites were distributed in 2406 unique genes. Based on our filtering criteria, all of these editing sites were located in unique genomic positions and were not close to any splice junction, bidirectional transcription, or low-complexity regions (such as SSRs). A summary of the statistics of raw and clean reads and mapping information as well as the number of identified RDDs and editing sites in different samples is provided in S1 File.

### 3.2. Sequence Preference Analysis

**Figure 4:**
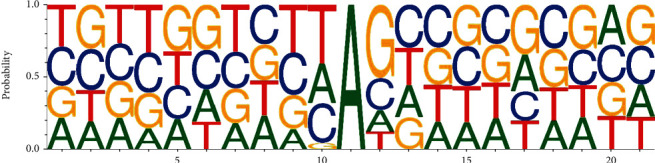
Neighborhood sequence preferences of nucleotides for RNA editing sites.

ADAR enzyme targets dsRNA of any sequence, but it has a sequence preference in the vicinity of the editing sites. Consistent with the known attributes of ADAR substrates, our results showed that the nucleotide immediately upstream (relative -1 location) of the edited site had a strong preference for G depletion and T enrichment, while the nucleotide immediately downstream (relative +1 location) of the editing site showed significantly depleted T and favored G (see Figure 4).

### 3.3. Validation of Identified Editing

**Figure 5:**
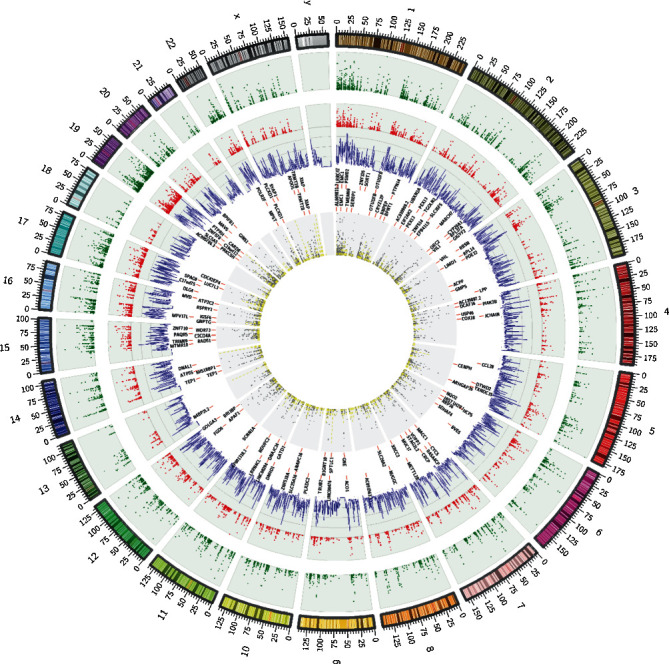
Profiling of RNA editing sites in normal and cancer tissues of gastric cancer patients. The human genome is represented as the outermost ring. Each of normal and cancer tissue editing sites is shown by green and red dots. The purple line plot indicates Alu repeat distribution across the genome. Also, outer and inner text circles indicate normal-specific differentially edited genes and cancer-specific differentially edited genes, respectively. Yellow bars represent microRNA targeting sites in the genome, and grey scatter dots indicate editing sites in these regions.

**Figure 6:**
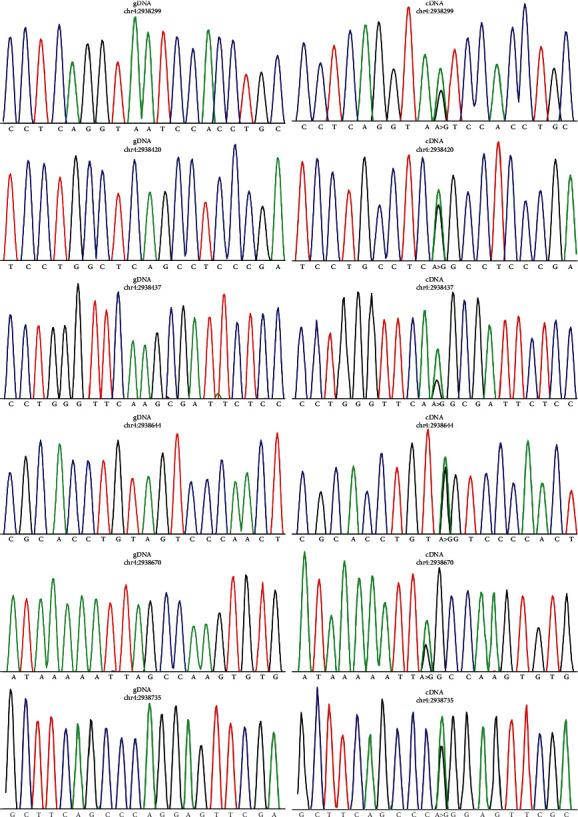
Chromatograms of the sequences of gDNA and cDNA at six editing loci of NOP14.

**Figure 7:**
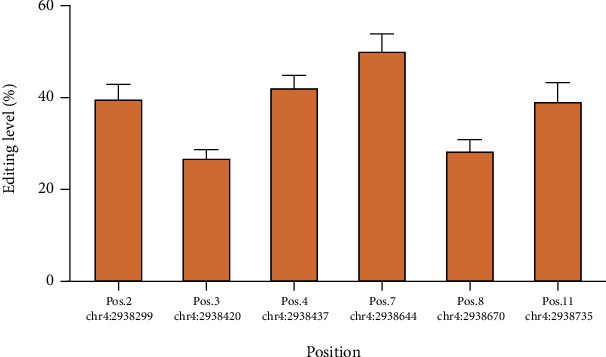
Average editing level in six editing loci of NOP14.

The location of identified editing sites was compared with the position of Alu elements across the genome. Interestingly, distribution of A to G editing sites and Alu elements was very similar across the genome. This is more obvious when we look closely at chromosomes 1, 9, 16, and 19, where ends of these chromosomes are rich in Alu repeats but the middle of chromosomes are relatively vacant (see Figure 5). Next, to validate whether the identified RNA editing sites were true positive, we searched for evidence of the identified RNA editing sites in expressed sequence tags (ESTs) based on the NCBI database. Of 12105 known and 257 novel editing sites, 10944 (90.4%) and 218 (84.8%) sites were found in EST clones, respectively. Further investigation revealed that 7643 (68.5%) of the identified editing events were validated in more than five EST clones, which reinforce the accuracy of our method. Indeed, for experimental validation of identified editing sites, we selected 11 sites on the NOP14 gene. Six of these editing sites (positions 2, 3, 4, 7, 8, and 11) were validated through Sanger sequencing (Figure 6), but we failed to identify the other five editing sites. The average editing level in each position has been shown in Figure 7.

### 3.4. Distribution of the Editing Sites across Genomic Regions

**Figure 8:**
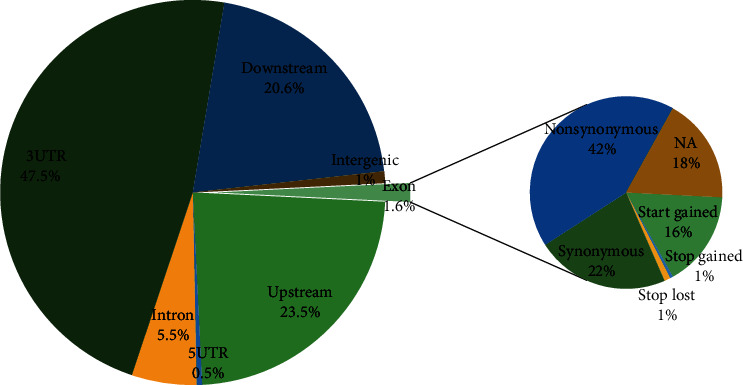
Distribution of RNA editing sites in different genomic regions.

The location of editing sites was annotated according to the Ensembl database. As shown in Table 1, the most frequent biotype of edited transcripts was “protein coding” and the least was “snoRNA.” Also, 42 editing sites were located in miRNAs, which belonged to 17 unique microRNAs. Of these, the MIR34A precursor included 10 cancer-specific editing sites (Table 2). Investigation of the genomic distribution of editing sites showed that the number of RNA editing sites was greatly varied across genomic regions. Overall, the 3′UTR was the most edited region, with 5870 editing sites (45.5% of all detected editing sites), followed by the upstream region (23.5%), and the 5′UTR had the least number of editing sites (less than 1%). Indeed, 192 (1.6%) of the editing sites were located in exons, including 81 sites (42% of exonic editing sites) with nonsynonymous effect and 43 sites (22%) with synonymous effect. Exonic RNA editing leads to at least one premature termination codon in the RPL9 (chr4:39456431) gene and two stop loss mutations in ZSWIM7 (chr17:15986849) and NEIL1 (chr15:75351324) genes. Also, one editing site with start-gain mutation effect was detected (see Figure 8).

### 3.5. Frequency of Editing Sites and RNA Editing Level

**Figure 9:**
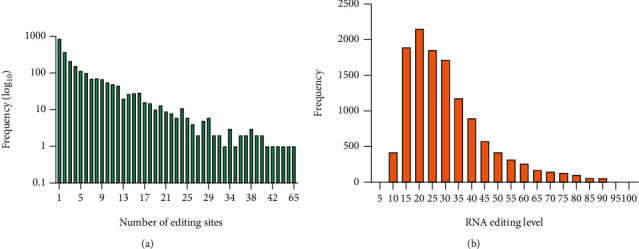
Frequency histogram of the number of editing sites (a) and frequency distribution plots of RNA editing levels (b).

RNA editing sites often appear in clusters, due to simultaneous editing of multiple adenosines by ADAR proteins. In this respect, we investigated whether the identified editing sites were in clusters or not. We found that 34% of genes were edited in more than five sites. Furthermore, the frequency of editing sites (number of edited sites located in the gene) was calculated to evaluate clustering of editing sites. Our results showed an editing rate of 5.1, indicating on average five editing sites per gene. Interestingly, editing rates were different when genomic regions were considered separately. The editing rate in 3′UTRs was predominant, 7.1 editing sites per gene, and exons with 1.4 editing sites per gene exhibited the least editing rate. The editing rate in upstream and downstream regions, which included a large number of editing sites, was 3.5 and 3.8 sites per gene, respectively. The frequency of the number of editing sites across genomic regions is shown in Figure 9(a). RNA editing level was also calculated for all edited sites, using the following formula [53]:

(1) $$\begin{array}{c} \text{RNA editing level}=\frac{\text{number of reads supporting edited allele}\times 100}{\text{total number of reads at a site}}. \end{array}$$

The average RNA editing level across all sites was 30.72, which means that approximately 31% of each gene transcript was edited in a given site. In the present study, editing level for most of the identified editing sites ranged from 15 to 25. The frequency distribution of RNA editing levels is shown in Figure 9(b).

### 3.6. Association between Chromosome Length, Alu Elements, Protein Coding Genes, and Number of Editing Sites

**Figure 10:**
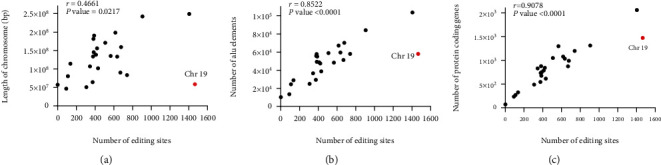
Association between the number of editing sites and (a) length of the chromosome, (b) number of Alu elements, and (c) number of protein coding genes.

Spearman's correlation coefficient was used to investigate the association between the number of editing sites and the length of chromosomes. As expected, the number of RNA editing sites tended to be associated with chromosome length, but the association was weak when all chromosomes were included (*r* = 0.47, *P* = 0.02). As shown in Figure 10(a), chromosome 19 has the highest editing frequency according to its size. Excluding chromosome 19, the analysis showed a significant correlation between the number of RNA editing sites and length of chromosomes (*r* = 0.6, *P* < 0.002). In addition, the correlation of editing with both the number of Alu elements and the number of protein coding genes were calculated. Surprisingly, we found that correlation of editing with the number of protein coding genes was stronger than the number of Alu elements, where Spearman's correlation coefficient was 0.91 and 0.85, respectively (Figures 10(b) and 10(c)). For further investigation, we calculate the editing rate for each chromosome as the number of editing sites in one kilobase (kb). Our results showed that chromosome 19 has the most rate of editing with one editing site per 40 kb, followed by chromosome 17 with one editing site per 112 kb. On average, one editing site was identified in 250 kb of the human genome, and gene-poor chromosomes (18, 4, 21, 13, and Y) have the least rate of editing (S2 File).

### 3.7. Cancer- and Normal-Specific Editing Sites

**Figure 11:**
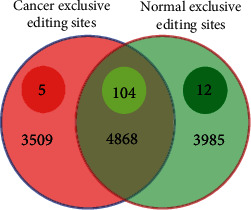
Number of editing sites in cancer and normal tissues. Inner circles indicate the number of differentially edited sites between two groups (*P* value < 0.05).

Among the 12362 editing sites, 4868 sites were found within both normal and cancer samples. From the remaining sites, 3985 and 3509 editing sites were specific to normal and cancer tissues, respectively. Statistical analysis revealed 104 differentially edited events among common editing sites. Notably, 5 cancer-specific and 12 normal-specific editing sites were found to be differentially edited (see Figure 11).

### 3.8. Functional Impacts of RNA Editing Sites

The functional impact of RNA editing could induce by a vast range of molecular mechanisms. For instance, it can lead to amino acid recoding, causing changes in seed sequences of microRNAs or affecting microRNA targeting sites. In search of amino acid recoding mutations, 81 editing sites were found across 63 genes that could lead to nonsynonymous change (S3 File), including 12 novel editing sites. Interestingly, MUC4, an epithelial glycoprotein coding gene, was edited in two positions (3:195780295 and 3:195780902), which caused p.L3762P and p.S2560P, respectively (Table 3). MicroRNA targeting would be affected by editing. In this regard, 44 editing sites were detected that affect microRNA target recognition in normal and tumor tissues of gastric cancer (Table 4). In addition, 294 editing sites with non-sense-mediated decay impact were found that affect 92 protein coding genes. Of these, 80 and 111 sites were identified only in cancer and normal samples, respectively. Also, 103 non-sense-mediated decay editing sites were found in both cancer and normal tissues (S4 File).

## 4. Discussion

The identification of RNA editing sites deeply depends on sequencing technology and bioinformatics approaches. We developed a pipeline for identifying RNA editing events in primary gastric cancer and normal tissues by screening RNA differences from the reference genome followed by successive and rigorous filtering criteria. Some of the previous studies have applied coupled RNA and DNA sequences to identify editing events [29, 54]; however, we identified RNA editing sites using solely RNA sequencing data. Our analyses found a significant number of editing sites, and the vast majority of them were harbored in 3′UTR regions, as previously reported [53, 55]. A few novel editing sites were also found, which were reported for the first time in our study. Although the number of identified RNA editing sites was huge, most of the sites exhibited low editing levels and approximately half of the identified sites were edited in less than 27% of their related transcripts.

Our analyses found that the RNA editing sites were highly associated with both the number of protein coding genes and Alu element distribution in the genome. The frequency of editing sites was correlated with the size of chromosomes. These results are in good agreement with Chigaev et al.'s study who reported that correlation of editing frequency with protein coding genes was stronger than lincRNA density [53]. However, this correlation could result from the bias of the library preparation step of RNA sequencing projects. Since oligo-dT primers were applied to capture the RNA through the poly-A tail, most of the reads would be related to protein coding genes.

To date, no specific sequence has been found that characterizes editing sites of any of the ADAR enzymes. However, in the neighborhood of edited adenosine, there are preferred and opposed preferences. Consistent with previous studies, there was an overrepresentation of guanosine in the downstream position of the editing site, while guanosine was depleted in the upstream neighboring position [27, 55]. Since some of the adenine bases in the right context are not edited, other features were proposed to be involved in the determination of editing. Daniel et al. described editing inducer elements distant from the edited adenine, which promote the editing efficiency and specificity of a highly edited site [21]. Wong et al. reported that editing efficiency is strongly influenced by the base opposing the edited adenosine. They found that when there was an A:C mismatch at the editing site, editing by the ADAR enzyme was enhanced compared to A:A or A:G mismatches or A:U at the same site [22]. Due to the contradictory results, it is difficult to make definitive conclusions about potential editing sites.

We wonder whether RNA editing could function as an additional mechanism contributing to tumorigenesis by generating specific RNA editing sites that were unique to cancer samples. In search of the answer to this question, we found that 28.4% and 32.2% of the identified editing sites were specific to cancer and normal tissues, respectively. These tissue-specific editing sites could contribute to cancer initiation and progression, if they are located in an important gene. Some of cancer-specific editing sites and their role in the pathogenesis of cancer have been identified in previous studies. RNA editing of the transcription factor PROX1, a candidate tumor suppressor, leads to several missense substitutions including E328G, R334G, and H536R and loses tumor suppressive functions. These editing events have been seen in a number of esophageal, pancreatic, and colon cancer samples, but not in cDNA libraries of many normal tissues [16].

We also found a remarkable number of common editing events between cancer and normal tissues, in which their editing levels were significantly different. Deregulated editing level in cancer and normal common editing sites could be an important contributor in tumorigenesis. Chen et al. reported that RNA editing level of AZIN1 was increased by at least 10% in hepatocellular carcinoma compared to the adjacent normal liver. The edited isoform compared with wild-type AZIN1 has increased affinity to antizyme, which leads to neutralization of antizyme-mediated degradation of ornithine decarboxylase and cyclin D1 and promotes cell proliferation [56]. In this regard, Han et al. reported a higher level of editing on RHOQ in a tumor compared with normal tissue in colorectal cancer, which results in N136S amino acid substitution. This RNA mutation increases RHOQ protein activity, actin cytoskeletal reorganization, and invasion potential [57]. On the contrary, hypoediting of several genes is associated with cancer phenotypes. The pre-mRNA transcript encoding the GluR-B has two functionally important editing sites (Q/R and R/G sites), and the Q/R site is almost entirely edited, which is a necessity for the normal function of the receptor. It has been proven that in malignant tissue of human brain tumors, this editing site of GluR-B considerably is underedited compared with that in control tissues [58]. Our results corroborate that the RNA editing level could be differentially regulated in tumor and normal tissues, which is consistent with observations reported previously.

Our results showed that the vast majority of editing sites in gastric cancer were located in 3′UTR and up/downstream regions as well as in coding regions. According to their genomic location, these RNA editing events could lead to various functional impacts and apply their effects through several dominant mechanisms. First and most important, RNA editing events in the exonic region can cause amino acid change and imitate cancer-associated missense mutations. Our pipeline identified 81 editing events with nonsynonymous effect, including 12 novel editing events. Notably, we found four missense RNA mutations in the mucin family (MUC3A, MUC4, and MUC6). Normal gastric epithelial cells transcribe MUCs, which have several functions including protection against mechanical and infectious lesions, lubrication, and acid resistance [59]. Several studies have reported that the transcription profile of mucins is changed in gastrointestinal cancers, which overall suggests an important role for MUCs in gastric cancer [60–62]. Our results reinforced the hypothesis that inappropriate RNA editing can be involved in gastric cancer development.

Second, RNA editing could affect microRNA target recognition and subsequently affect the expression profile of the genes. Dysregulation of microRNA target recognition has been linked to cancers [63, 64]. In this context, 44 editing events were found in the present study, where at least one microRNA binding was disrupted. Consistent with our research, Soundararajan et al. identified 652 editing events in lung cancer, which were located in the 3′UTR of 205 target genes and mapped to 932 potential microRNA target binding sites [65]. Altogether, these findings are inconsistent with Liang and Landweber's computational analyses, where they suggested that RNA editing mainly avoids microRNA target sites, even though RNA editing events potentially block the microRNA target recognition sites [66]. It is worth reminding that RNA editing events in addition to disrupting existing microRNA binding sites could generate novel microRNA regulatory networks. In a completely separate mechanism from what has been mentioned, RNA editing could affect microRNA biosynthesis. miR-142 is highly expressed in hematopoietic tissues, but not in nonhematopoietic tissues. Its expression in patients with acute myeloid leukemia is significantly lower than that in controls. Yang et al. showed that editing of pri-miR-142 leads to suppression of its processing by Drosha and subsequently its degradation [67].

Third, the editing of microRNA sequences could alter their binding affinity or target recognition properties. Since microRNAs play a role in nearly all cellular pathways and pathological processes, including cancer initiation and progression, fluctuations of their targeting are an important contributor to cancer [68]. Our analysis revealed 42 editing sites in 17 cancer-associated microRNAs, and some of them are exclusively edited in cancerous tissue. Consistent with our results, Nigita et al. identified 40 and 18 potential editing sites in lung adenocarcinoma and lung squamous cell carcinoma, respectively [69]. Our results showed miR-34a, a cancer-specific edited microRNA, was edited in 10 positions. Previous studies have identified this microRNA as a tumor suppressor in gastric cancer cell lines [70]. On the other hand, it was shown that miR-34a was epigenetically downregulated or silenced in gastric cancer tissues and cell lines [69]. We therefore speculate that editing in some positions could terminate the function of miR-34a, but further studies are required to confirm this possibility.

We consider a candidate gene with several editing sites and experimentally validated some of these sites. However, it should be noted that validating a small number of editing sites in 42 clinical samples is a limitation of the study which warrants further investigation and our validation strategy was biased towards a specific gene.

Findings of the current study uncovered a relatively large number of RNA editing sites, which were unevenly distributed across the genome. Editing level of these sites and editing rate of different genes had diverse distribution. We also found a significant number of exclusively edited genes in cancer and normal tissues, which are likely to contribute to cancer initiation and progression.

## 5. Conclusions

Gastric cancer initiation and progression are driven by the cumulative effects of genetic and epigenetic alterations; RNA editing, a widespread posttranscriptional mechanism, could be part of these alterations. Depending on genomic location and level of editing, this phenomenon could lead to missense mutations, affecting microRNA biosynthesis and targeting, changing splicing patterns, and modifying microRNA target sites. The editome of gastric cancer vastly differs from that of adjacent tissue in terms of both type and number of editing sites. Given the distinct pattern of RNA editing between gastric cancer and normal tissues, edited sites have the potential to serve as biomarkers and therapeutic targets in gastric cancer.

## Figures and Tables

**Table 1:** Number of different edited biotypes.

Biotype	No.
Protein coding	9000
snRNA	32
Processed transcript	893
Retained intron	996
lincRNA	347
Antisense	453
miRNA	42
Sense intronic	116
Sense overlapping	24
Pseudogene	212
TEC	63
snoRNA	12
miscRNA	21
Intergenic	122
NA	29

**Table 2:** List of edited microRNAs in gastric cancer and normal tissues.

Symbol	Chr.	Specification	No. of editing sites	Role in cancer
miR-1205	Chr8	Cancer	2	[71, 72]
miR-143	Chr5	Normal	6	[73–75]
miR-24-1	Chr9	Normal	1	[76, 77]
miR-3176	Chr16	Cancer	3	[78, 79]
miR-34A	Chr1	Cancer	10	[70, 80, 81]
miR-4315-2	Chr17	Cancer	1	[82]
miR-4522	Chr17	Cancer	1	[83]
miR-4539	Chr14	Common	1	[84]
miR-4728	Chr17	Cancer	2	[85, 86]
miR-559	Chr2	Normal	1	[87, 88]
miR-5692C2	Chr7	Normal	2	[89]
miR-612	Chr11	Cancer	1	[90, 91]
miR-621	Chr13	Normal	3	[92, 93]
miR-635	Chr17	Common	3	[94, 95]
miR-642B	Chr19	Normal	3	[96, 97]
miR-650	Chr22	Common	1	[98, 99]
miR-8071-1	Chr14	Common	1	[100]

**Table 3:** List of novel editing sites with nonsynonymous change.

Position	Gene ID	Editing effect
1: 246885532	AHCTF1	p.N883S
7: 142529491	TRBV7-9	p.N26D
17: 2333110	TSR1	p.S386G
11: 130914721	SNX19	p.S407G
17: 31856838	COPRS	p.S43G
11: 1018295	MUC6	p.I1502M
X: 315276	GTPBP6	p.I171V
7: 100958135	MUC3A	p.M2119T
3: 58156064	FLNB	p.M2324V
3: 195780295	MUC4	p.L3762P
3: 195783902	MUC4	p.S2560P
22: 22376266	IGLV5-45	p.C44R

**Table 4:** List of editing sites that affect microRNA target recognition.

Chr.	Position	Gene	Specify	Affected microRNA(s)
chr1	9100841	GPR157	Common	miR-490-3p
chr1	10459831	DFFA	Common	miR-150/5127
chr1	10460010	DFFA	Common	miR-208ab/208ab-3p
chr1	10460010	DFFA	Common	miR-499-5p
chr1	179073347	FAM20B	Common	miR-125a-5p/125b-5p/351/670/4319
chr1	179073347	FAM20B	Common	let-7/98/4458/4500
chr1	179075081	FAM20B	Normal	miR-22/22-3p
chr1	179075107	FAM20B	Normal	miR-146 ac/146b-5p
chr1	179075144	FAM20B	Normal	miR-143/1721/4770
chr4	2839669	SH3BP2	Cancer	miR-199ab-5p
chr4	2840078	SH3BP2	Common	miR-217
chr4	2840078	SH3BP2	Common	miR-200bc/429/548a
chr4	2938644	NOP14	Common	miR-24/24ab/24-3p
chr4	17626928	MED28	Normal	miR-455-5p
chr4	17632277	FAM184B	Cancer	miR-144
chr5	34906645	RAD1	Common	miR-143/1721/4770
chr5	37290314	NUP155	Common	miR-24/24ab/24-3p
chr5	43377383	CCL28	Normal	miR-383
chr5	43380635	CCL28	Common	miR-24/24ab/24-3p
chr5	75378054	COL4A3BP	Cancer	miR-103a/107/107ab
chr6	53100576	FBXO9	Normal	miR-103a/107/107ab
chr7	44802500	PPIA	Common	miR-22/22-3p
chr7	100212870	CASTOR3	Common	miR-128/128ab
chr7	100212870	CASTOR3	Common	miR-27abc/27a-3p
chr8	41542121	GINS4	Common	miR-26ab/1297/4465
chr8	43029279	HOOK3	Common	miR-26ab/1297/4465
chr8	43029280	HOOK3	Cancer	miR-26ab/1297/4465
chr9	128305442	TRUB2	Common	miR-15abc/16/16abc/195/322/424/497/1907
chr9	128305442	TRUB2	Common	miR-103a/107/107ab
chr11	768850	GATD1	Common	miR-141/200a
chr11	769393	GATD1	Cancer	miR-24/24ab/24-3p
chr11	111728428	SIK2	Normal	miR-142-3p
chr14	21460342	RAB2B	Common	miR-196abc
chr16	66887684	PDP2	Common	miR-7/7ab
chr19	1032835	CNN2	Common	miR-25/32/92abc/363/363-3p/367
chr19	1777958	ONECUT3	Common	miR-142-3p
chr19	1778139	ONECUT3	Common	miR-194
chr19	1778241	ONECUT3	Common	miR-218/218a
chr19	1778303	ONECUT3	Common	miR-103a/107/107ab
chr19	2835224	ZNF554	Normal	miR-17/20ab/20b-5p/93/106ab/427/518a-3p/519d
chr19	4653661	TNFAIP8L1	Common	miR-150/5127
chr19	16631220	SMIM7	Cancer	miR-192/215
chr19	18368012	PGPEP1	Common	miR-455-5p
chr20	3869487	MAVS	Normal	miR-338/338-3p

## Data Availability

The datasets that were analyzed in this study are publicly available through the GEO database with accession number of GSE85465. All other remaining data are available within the article and supplementary files or available from the authors upon request.

## Supplementary Materials

Supplementary 1 Summary of the statistics of raw and clean reads and mapping information.

Supplementary 2 Editing rate for each chromosome.

Supplementary 3 List of editing events with recoding impact.

Supplementary 4 Editing events with non-sense-mediated decay impact.
